# A fruit diet rather than invertebrate diet maintains a robust innate immunity in an omnivorous tropical songbird

**DOI:** 10.1111/1365-2656.13152

**Published:** 2020-01-03

**Authors:** Chima J. Nwaogu, Annabet Galema, Will Cresswell, Maurine W. Dietz, B. Irene Tieleman

**Affiliations:** ^1^ Groningen Institute for Evolutionary Life Sciences University of Groningen Groningen The Netherlands; ^2^ School of Biology University of St Andrews St Andrews Fife UK; ^3^ A.P. Leventis Ornithological Research Institute Jos Nigeria; ^4^ Present address: Fitzpatrick Institute of African Ornithology University of Cape Town Rondebosch 7701 Cape Town South Africa

**Keywords:** environmental change, immunomodulation, life‐history trade‐offs, nutrient limitation, path analyses, vegetarians

## Abstract

1. Diet alteration may lead to nutrient limitations even in the absence of food limitation, and this may affect physiological functions, including immunity. Nutrient limitations may also affect the maintenance of body mass and key life‐history events that may affect immune function. Yet, variation in immune function is largely attributed to energetic trade‐offs rather than specific nutrient constraints.
2. To test the effect of diet on life‐history traits, we tested how diet composition affects innate immune function, body mass and moult separately and in combination with each other, and then used path analyses to generate hypotheses about the mechanistic connections between immunity and body mass under different diet compositions.
3. We performed a balanced parallel and crossover design experiment with omnivorous common bulbuls *Pycnonotus barbatus* in out‐door aviaries in Nigeria. We fed 40 wild‐caught bulbuls ad libitum on fruits or invertebrates for 24 weeks, switching half of each group between treatments after 12 weeks. We assessed innate immune indices (haptoglobin, nitric oxide and ovotransferrin concentrations, and haemagglutination and haemolysis titres), body mass and primary moult, fortnightly. We simplified immune indices into three principal components (PCs), but we explored mechanistic connections between diet, body mass and each immune index separately.
4. Fruit‐fed bulbuls had higher body mass, earlier moult and showed higher values for two of the three immune PCs compared to invertebrate‐fed bulbuls. These effects were reversed when we switched bulbuls between treatments after 12 weeks. Exploring the correlations between immune function, body mass and moult, showed that an increase in immune function was associated with a decrease in body mass and delayed moult in invertebrate‐fed bulbuls, while fruit‐fed bulbuls maintained body mass despite variation in immune function. Path analyses indicated that diet composition was most likely to affect body mass and immune indices directly and independently from each other. Only haptoglobin concentration was indirectly linked to diet composition via body mass.
5. We demonstrated a causal effect of diet composition on innate immune function, body mass and moult: bulbuls were in a better condition when fed on fruits than invertebrates, confirming that innate immunity is nutrient specific. Our results are unique because they show a reversible effect of diet composition on wild adult birds whose immune systems are presumably fully developed and adapted to wild conditions—demonstrating a short‐term consequence of diet alteration on life‐history traits.

## Graphical abstract

The authors results confirm that innate immunity is nutrient specific, but they are unique because the authors show an experimentally reversible effect of diet composition on wild adult birds whose immune systems are presumably fully developed and adapted to wild conditions—demonstrating a short‐term consequence of diet alteration in natural systems.

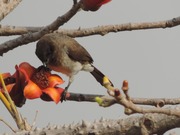

## 1 INTRODUCTION

Animals face nutrient limitations during their life or during the annual cycle, and such nutrient limitations may affect physiological and life‐history functions, including immunity (Klasing, 2007), body mass maintenance (Krieger, Sitren, Daniels, & Langkamp‐Henken, 2006) and key life‐history events (Cotter, Simpson, Raubenheimer, & Wilson, 2011), even in the absence of food limitation. Many annual omnivores are seasonal diet specialists, even showing changes in their digestive systems to accommodate diet shifts (McWilliams & Karasov, 2001; Piersma et al., 1999). Such shifts in diet may be associated with seasonal limitations in nutrients required for optimal immune function. For example, protein‐rich diets like insects may support leukocyte, antibody and acute phase protein synthesis (Mabuchi & Frankel, 2016), but may be poor in antioxidants (Griffiths et al., 2016), which play immuno‐modulatory and antioxidant functions (Isaksson, 2015; Klasing, 2007). Fruits and vegetables on the other hand are poor in proteins but rich in fibre, sugars, vitamins, flavonoids and carotenoids. Diet components also influence the gut microbiome (David et al., 2013; Pan & Yu, 2014) and this may impact immune function (Macke, Tasiemski, Massol, Callens, & Decaestecker, 2017). Nonetheless, it is difficult to predict which diet is best for immune function because different diets lack different nutrients (Cotter et al., 2011), and the immune system has different physiological pathways (Schmid‐Hempel & Ebert, 2003), but we may expect protein‐rich diets to be better because they contain essential amino acids that are not synthesized de novo.

How nutrient limitations modulate the relationship between immune function and other life‐history traits such as body mass maintenance and moult in birds is not well known (but see Pap, Vágási, Czirják, & Barta, 2008; Pap et al., 2009, 2011). Regulation of body mass and moult in birds are crucial aspects of self‐maintenance, especially for long‐lived species (Williams, 1966) and like immune function, these are subject to nutrient availability (Murphy, 1996; Murphy & Taruscio, 1995). Dietary protein supply is crucial for the maintenance of body form and function because there is no storage form of protein in the body, and this implies that protein limitation may lead to breakdown of fat‐free tissues (Krieger et al., 2006), including skeletal muscles and digestive organs (Piersma & Gill, 1998). Annual moult involves substantial tissue replacement and so moult may exploit tissue proteins if dietary supplies are limited (Podlaszczuk, Włodarczyk, Janiszewski, Kaczmarek, & Minias, 2017), especially because feather growth occurs during the post‐absorptive state (Chen et al., 2015). However, although feather keratin requires high amounts of sulphur amino acids (methionine and cysteine) to synthesize (Murphy & King, 1984), the ‘actual nutrient requirements’ of moult are unclear because moult may proceed with limited nutrients or under a wide variety of diets (Murphy & King, 1992), albeit resulting in poor quality feathers or protraction of the moulting period (Murphy, King, & Lu, 1988; Vágási, Pap, & Barta, 2010).

**Figure 1:**
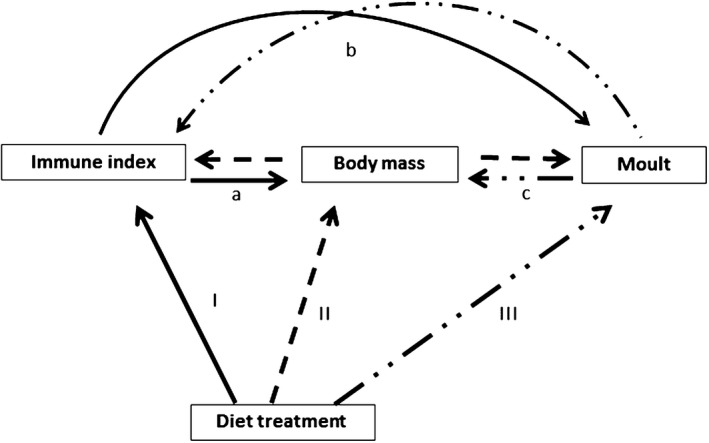
Diet composition may affect immune function, body mass and moult directly (I, II & III). Immune indices, body mass and moult may covary (a, b and c) if diet affects all or any of immune function, body mass or moult with resultant effect on others. Diet effect may follow alternative indirect pathways: solid lines—diet affects immune index and immune index affects body mass and/or moult, or dashed lines—diet affects body mass, body mass affects immune index and/or moult, or dotdash lines—diet affect moult and moult affects body mass and/or immune index. Note however, that the hypothetical indirect effect of diet which arises from a direct effect on moult can only be tested reasonably in the moulting season, and so was not considered

Immune function, body mass and moult may covary if they exploit similar nutrients, especially if such nutrients are in short supply (Figure 1a–c). Thus, exploring such covariations under different diet composition will aid the formulation of hypotheses that test how diet alteration and resultant nutrient limitations affect immune function, body mass and moult via potential trade‐offs in natural systems (Sanz, Moreno, Merino, & Tomás, 2004). But there are further complications to understanding variation in immune indices because of our limited knowledge of the mechanistic connections between life‐history traits (Hegemann, Matson, Flinks, & Tieleman, 2013a). Diet alteration can affect several linked processes. For example, diet effects on immune function and moult may arise from its prior effect on body mass (dashed lines, Figure 1), or diet effects on body mass and moult may arise from its prior effect on immune function (solid lines, Figure 1). A prior effect of diet on moult may also affect body mass and possibly immune function (dotdashed lines, Figure 1), but this may be restricted to the moulting period in seasonally moulting birds. Poor nutrition, body mass loss and susceptibility to infection are intricately linked (Beldomenico et al., 2008; Schaible & Kaufmann, 2007) and these may affect different immune indices differently, but, it still remains equivocal whether high immune indices are good or bad (Adamo, 2004; Matson, Cohen, Klasing, Ricklefs, & Scheuerlein, 2006). Thus, by using path analyses to explore connections between immune indices and body mass, we can generate hypotheses on mechanisms linking immune indices and other life‐history traits. Path analyses generate hypotheses about causal connections based on correlative datasets (Shipley, 2009). This knowledge is lacking, yet crucial for interpreting immune indices in light of individual fitness.

Omnivorous birds such as common bulbuls *Pycnonotus barbatus* are good models for testing the effect of diet composition on innate immune function, and more broadly, for understanding how specific immune indices associate with other life‐history traits during nutrient rather than food limitations. Some birds switch diets seasonally (Bairlein, 1996; Jenni‐Eiermann & Jenni, 2003; Kissling, Sekercioglu, & Jetz, 2012; Marshall, Dick, & Guglielmo, 2016), but common bulbuls are year‐round omnivores (Nwaogu, 2019). They feed predominantly on fruits and invertebrates, and occasionally on nectar and seeds (Milla, Doumandji, Voisin, & Baziz, 2005; Okosodo, Obasogie, & Orimaye, 2016). By restricting common bulbuls to fruits or invertebrates only, we can explicitely identify their effects on immune function and other life‐history traits.

Here we test how fruit and invertebrate diets affect innate immune function, body mass and extent of primary moult of wild‐caught captive common bulbuls. First, we compared innate immune indices, body mass and extent of primary moult among and within common bulbuls fed on fruits or invertebrates, or switched between treatments after 12 weeks. We made comparisons before diet restriction, 12 weeks later on switching, and after a further 12 weeks after switching treatments. We predicted that: (a) birds on protein‐rich invertebrate diet will maintain higher body mass, earlier moult and higher innate immune indices. Secondly, we compared patterns of covariation between immune function, body mass and extent of moult for fruit and invertebrate‐fed bulbuls. We expected that: (a) nutrient limitations due to diet manipulation will lead to negative correlations between immune response, body mass and extent of primary moult and this should be more pronounced in protein‐deprived fruit‐fed bulbuls. Finally, we used path analyses to generate hypothetical pathways to explain covariations between immune indices and body mass. We explored whether the diet effect on immune function and body mass is direct (I & II, Figure 1) or via two alternative indirect pathways: (a) the diet effect on body mass is via a diet effect on immune function (solid lines, Figure 1) or (b) the effect of diet on immune function is via a diet effect on body mass (dashed lines, Figure 1). We could not test for the indirect effect of diet on immune function and body mass via moult (dot‐dashed lines Figure 1), because the extent of moult was measured only once at the end of the experiment.

## 2 MATERIALS AND METHODS

### 2.1 Study species

Common bulbuls weigh 25–50 g. They are sexually monomorphic, but males are slightly bigger than females. They are resident in central Nigeria where they experience one wet and one dry season lasting about six months each annually. They breed year‐round but moult in the wet season (Nwaogu, Tieleman, & Cresswell, 2019). Common bulbuls forage on different plants following availability. Some of these plants are supported by gullies in riparian forest fragments which hold water in the dry season. This buffering of environmental conditions by riparian forest fragments and variable plant phenology supports year‐round omnivory in wild common bulbuls (Nwaogu *pers. ob*s.).

### 2.2 Experimental set‐up

We caught 40 adult common bulbuls using mist nets around the A. P. Leventis Ornithological Research Institute (APLORI) in Nigeria (09°52'N, 08°58'E) between 28 October to 7 November 2016 and housed them in groups of 10 birds in four adjacent out‐door aviaries at APLORI. The aviaries measured 3 × 1 × 2 m and consisted of a concrete floor, a metal frame, wire mesh and a thatched roof made from grass mats to provide shelter. Birds were fed fruits and invertebrates in captivity until the experiment started on the 2 December 2016. Birds were supplied water and food ad libitum before and throughout the experiment: extra food was provided if food was depleted before sun set and old food was removed and replaced with new food every morning. All birds were sampled for blood, assessed for moult and weighed to determine baseline body mass and innate immune function on 1 or 2 December, before diet restriction commenced on 2 December. During the experiment, birds in two aviaries were fed fruits (F), and the other two were fed invertebrates (I). We then sampled fortnightly. After 12 weeks of diet treatment, five birds from each aviary were switched between treatments, and the other five birds of each aviary remained on the same treatment. Switched birds replaced each other in aviaries with the alternative diet treatment, so we maintained four aviaries with the same diet treatment throughout the experiment. In one of the fruit treatment aviaries, we moved only four birds to the invertebrate treatment because we had nine birds left in this aviary. The experiment continued for another 12 weeks. Thus, we grouped individuals as: II‐invertebrate throughout, IF‐invertebrate to fruit, FI‐fruit to invertebrate and FF‐fruit throughout. There were six females and 14 males on fruit diet and nine females and 11 males on invertebrate diet at the start of the experiment, but we were blind to the sex of individuals during the experiment, because sexes were only determined molecularly after the experiment. See Table S1 for details of sample size, sex and the causes of differences in group sizes throughout the experiment.

### 2.3 Diet treatment

The invertebrate diet consisted of crushed air‐dried grasshoppers, rehydrated crayfish and mealworms, and was supplemented with live insects caught in the reserve using sweep nets every other day. The fruit diet consisted of fruits available to common bulbuls in the wild at different parts of the year (Nwaogu *pers obs*). When available we provided bulbuls with ripe fruits of *Lantana camara, Phyllanthus muellerianus, Bridelia ferruginea*, *Harungana madagascariensis, Rhus natalensis, Jasminum dichotomum* and *Santaloides afzeli,* and supplemented these with chopped ripe papaya fruits daily. Both treatment groups were provisioned ad libitum.

### 2.4 Blood sampling, body mass and sexing

Birds were sampled between 6:00 and 10:00 hr daily in two consecutive days per sampling session. Two aviaries of alternate diet treatments were sampled per day, with sampling order rotating between sampling sessions. Birds were caught and sampled randomly per room and returned together after the last bird was sampled from a room. Birds were held in soft dark cloth bags after capture from the aviary and after sampling to minimize stress. On average, each bird was bled within 6.04 ± 3.76 min of removal from the aviary, but because disturbance may commence from the first capture of the day, we also accounted for the time between the first capture and all subsequent captures each day. We collected *c.* 300 ml of blood from each bird into heparinized micro‐capillary tubes after puncturing the brachial vein with a needle. Samples were emptied into 1.5 ml Eppendorf tubes and stored on ice until processing in the laboratory to separate plasma from cellular fractions. Separation was done within 4 hr of sampling. Plasma and blood cells were stored at −20°C for one week and then moved to −80°C until transported for immune assays in Groningen, the Netherlands.

We weighed (±0.1 g, Ohaus Scout) each bird and assessed primary moult after blood sampling. Extent of primary moult was determined by scoring each primary feather on a scale of 0–5: newly full grown feathers were scored 5 while unmoulted old feathers were scored 0, and feathers at intermediate stages of growth were scored 1–4 (Ginn & Melville, 1983). Scores were summed up to obtain the cumulative score of primary feathers per individual.

All birds were sexed using gel electrophoresis. We extracted DNA following methods by Richardson, Jury, Blaakmeer, Komdeur, and Burke (2001) and performed PCR with the P2/P8 primers (Griffiths, Daan, & Dijkstra, 1996).

### 2.5 Immune assays

#### 2.5.1 Haptoglobin concentration

Haptoglobin is a positive acute phase protein which circulates in low concentration but increases with inflammation (Jain, Gautam, & Naseem, 2011; but see Hegemann, Matson, Versteegh, Villegas, & Tieleman, 2013b). Haptoglobin binds to and removes haem from circulation during infection, making haem unavailable to pathogens. We quantified plasma haptoglobin concentration using a functional colorimetric assay, which quantifies the haem‐binding capacity of plasma. We followed instructions for the ‘manual method’ provided with a commercially available assay kit (Cat. No.: TP801; Tridelta Development Ltd, Maynooth, Co. Kildere, Ireland) (Matson, Horrocks, Versteegh, & Tieleman, 2012). We calculated within‐assay variability (*n* = 6 plates, maximum CV = 0.61, minimum CV = 0.42, mean CV = 0.51) and among‐assay variability (*n* = 450 samples, CV = 0.51) to verify consistency.

#### 2.5.2 Nitric oxide concentration

Nitric oxide modulates inflammatory processes and participates in the direct killing of parasites and tumor cells (Sild & Hõrak, 2009). We measured nitric oxide concentration by a colorimetric assay described by Sild and Hõrak (2009). The method estimates the concentration of nitrate and nitrite in plasma after reducing all nitrate to nitrite using copper‐coated cadmium granules. A measurable colour development proportionate to nitric oxide concentration follows reaction with Griess reagent. We calculated within‐assay variability (*n* = 6 plates, maximum CV = 1.79, minimum CV = 1.16, mean CV = 1.49) and among‐assay variability (*n* = 443 samples, CV = 1.51) to verify consistency.

#### 2.5.3 Ovotransferrin concentration

Ovotransferrin is a negative acute phase protein. Like haptoglobin, it binds to haem during infection, but its concentrations may decrease with increased inflammation due to temporarily high free hormones bound to ovotransferrin or the increased production of other acute phase proteins (Giansanti, Leboffe, Pitari, Ippoliti, & Antonini, 2012; Gruys, Toussaint, Niewold, & Koopmans, 2005; Jain et al., 2011). Ovotransferrin was quantified by estimating the maximum amount of iron required to saturate all ovotransferrin in a sample. We followed a three‐step process described by Horrocks, Irene Tieleman, and Matson (2011): saturation of ovotransferrin with ferric iron under alkaline conditions, reduction of excess unbound iron by ascorbic acid, then dissociation of ovotransferrin–iron complex under acidic conditions, leading to a colour development whose absorbance is measured by colorimetry. We calculated within‐assay variability (*n* = 16 plates, maximum CV = 1.32, minimum CV = 0.30, mean CV = 0.63) and among‐assay variability (*n* = 427 samples, CV = 0.69) to verify consistency.

All colorimetric assays (a–c above) were carried out using a Versamax plate reader (Molecular Devices Sunnyvale).

#### 2.5.4 Haemagglutination/haemolysis titres

We assessed natural antibody‐mediated haemagglutination and complement‐mediated haemolysis titres of plasma samples against 1% rabbit red blood cells (Envigo RMS (UK) Ltd.) in phosphate buffered saline as described by Matson, Ricklefs, and Klasing (2005). Both haemagglutination and haemolysis titres were recorded as the number of serial dilution steps in which each function was still observable. Haemagglutination and haemolysis titres were scored blind to individual and treatment using an existing rubric (Matson et al., 2005). We calculated within‐assay variability (*n* = 75 plates, haemagglutination: maximum CV = 0.27, minimum CV = 0.01, mean CV = 0.10; haemolysis: maximum CV = 2.45, minimum CV = 0.22, mean CV = 0.76) and among‐assay variability (*n* = 450 samples, haemagglutination: CV = 0.13; haemolysis: CV = 0.72) to ensure consistency.

### 2.6 Statistical analyses

#### 2.6.1 Principal component analysis

We identified three principal components (PCs) with eigenvalues > 1 which cumulatively accounted for 66% of the total variation in immune indices (Table S2). Loading of the PC axes after varimax rotation revealed: PC1 (23.2%)—decreasing haptoglobin concentration and increasing haemolysis titre, PC2 (22.7%)—increasing ovotransferrin concentration and haemagglutination titre, and PC3 (20.0%)—increasing nitric oxide concentration (Table S2). About 34% of total variation in immune indices was unexplained by the PCs, but we used the PCs instead of raw immune indices, because some simplification was achieved. Repeating analyses with the original measures give very similar results.

##### Direct effect of diet composition on immune function, body mass and moult

To test the effect of diet treatment on immune function, body mass and moult, we built a general linear mixed effect model for each of the three principal components of immune indices, body mass and extent of primary moult. We included week, diet treatment and sex, and an interaction between week and diet treatment as main effects. For all principal components and body mass, we compared groups at week 0 and week 12 and between week 0 and 12 to test diet treatment effect, and between week 12 and 24 to confirm the effect after treatment switch. For moult, we only compared extent of primary moult at week 24 relative to week 12 because there were no birds moulting before week 12. Moult in common bulbuls starts on the 1st of May on average (Nwaogu, Tieleman, et al., 2019) and this was after week 12.

For all models above, we included individual identity nested in aviary as the random factor to account for individual variability and aviary effects. For models of the principal components of immune function and body mass, we also included the time lag between capture and sampling and that between the first capture of each day and the sampling of every bird to account for the effect of holding time on body mass and initial disturbance on immune indices.

##### Diet effects on covariation between immune function, body mass and moult

To compare covariation between body mass and innate immune function under fruit and invertebrate diets, we built three separate general linear mixed effect models, each with body mass as response to an interaction between diet treatment and a principal component of immune indices. We included week as main effect to account for the effect of temporal environmental factors (occurrence of rain and temperature variation [see Figure S1]) on body mass and immune indices irrespective of diet. We also included the time lag between capture and sampling and that between the first capture of each day and the sampling of each bird in all models to account for the effect of holding time on body mass and initial disturbance on immune indices. We used all data from week 0–24, assigning diet treatment as the diet of an individual prior to sampling (I or F), regardless of whether the individual was switched between diets or not. However, we accounted for individual variability, diet switch and aviary effects by nesting individual identity in the four‐level diet treatment (II, IF, FI and FF).

We tested for covariation between moult and immune indices at week 24 only, because common bulbuls were not moulting earlier. We built three separate general linear models with cumulative scores of primary feathers as response to an interaction between diet treatment and a principal component of immune indices. Similarly, we tested for covariation between moult and body mass at week 24. We built a general linear model with cumulative scores of primary feathers as response to an interaction between diet treatment and body mass. Again, we assigned diet treatment as the diet of an individual prior to sampling (I or F), regardless of whether individual was switched between diets or not.

##### Hypothetical pathways of diet effects on immune indices and body mass

To explore if diet composition affects immune function directly or indirectly through body mass, and—likewise—to explore if diet composition affects body mass directly or indirectly through immune function (Figure 1), we performed path analyses using the piecewiseSEM package in r (Lefcheck, 2015). For each immune index, we built two structural equation models, each in turn comparing two linear models with each other: Model A compared the alternatives that the effect of diet on immune function was direct (Model I, Figure 1) or through body mass (Model a (dashed line), Figure 1). Model B compared the alternatives that the effect of diet on body mass was direct (Model II, Figure 1) or through immune function (Model a (solid line), Figure 1). We then compared the relative fit of the alternative structural equation models (Model A and Model B) using AIC scores. We included individual identity nested in week as the random factor to account for individual and temporal variation.

## 3 RESULTS

### 3.1 Diet composition affects body mass, moult and innate immune function

#### 3.1.1 Innate immune function

**Figure 2:**
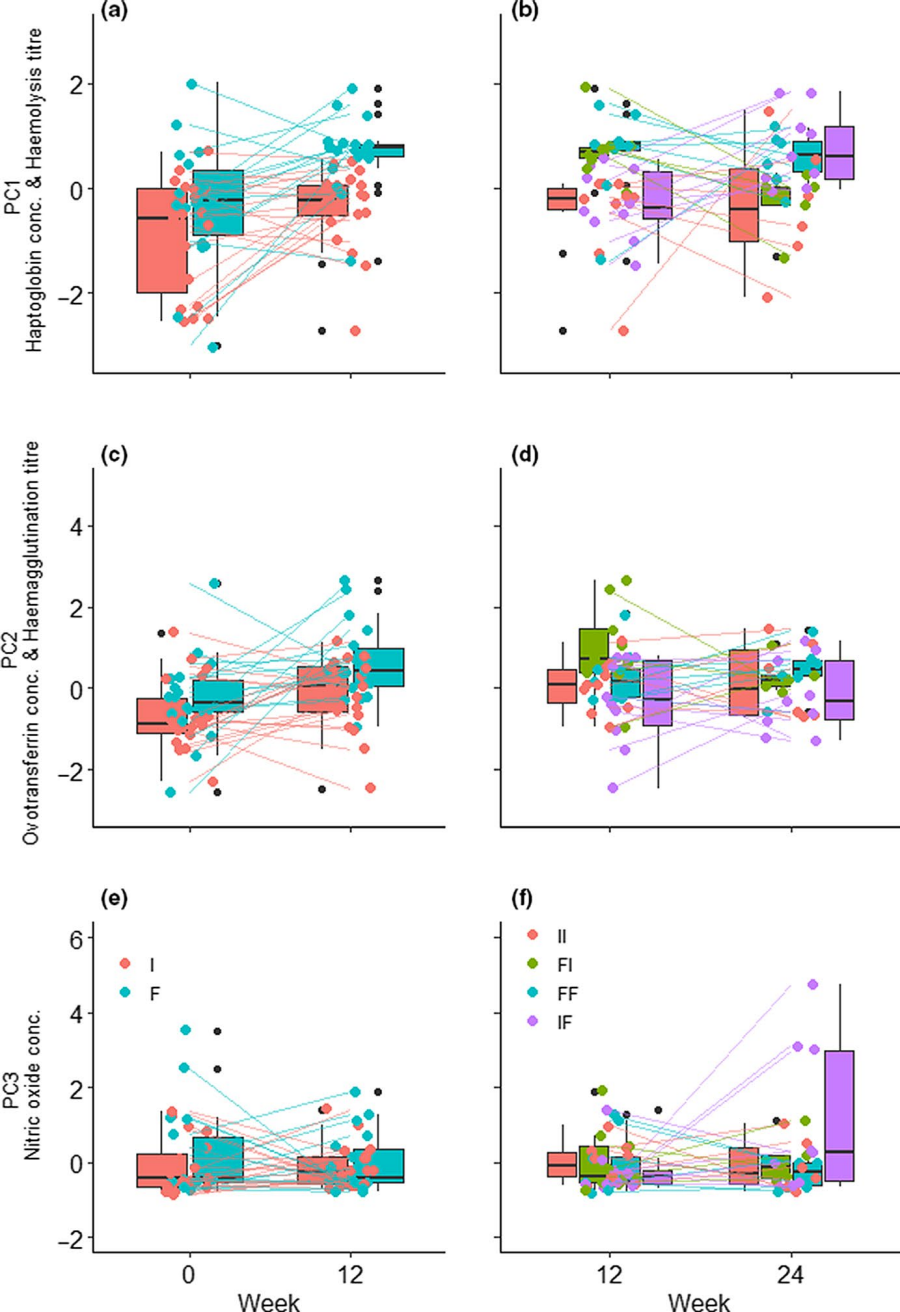
Effect of diet treatment on (a–b) PC1 (decreasing haptoglobin concentration and increasing haemolysis titre), (c–d) PC2 (increasing ovotransferrin concentration and haemagglutination titre) and (e–f) PC3 (increasing nitric oxide concentration) in the common bulbul. Bars show group medians while lines connect individuals. A subset of each treatment was switched to alternative diet treatment at week 12: FI‐fruit to invertebrate, IF‐invertebrate to fruit, FF‐ fruit throughout and II‐invertebrate throughout. Black dots are outliers from box plots while coloured dots are individual points

Measures of innate immunity did not differ between the diet treatment groups at the start of the experiment (Figure 2), however, at week 12, fruit‐fed bulbuls had significantly higher PC1 (decreasing haptoglobin/increasing heamolysis) than invertebrate‐fed ones (Tables 1 and 2). At week 24, treatment subsets did not differ significantly from each other (Table 2), however, the fruit‐fed subset that was switched to invertebrate diet decreased significantly in PC1 between weeks 12 and 24 (*F*_3, 53_ = 1.26, *p* = .02) while the subset that remained on fruit diet did not (*F*_3, 53_ = 0.14, *p* = .75). Accordingly, the invertebrate‐fed subset switched to fruit diet increased significantly in PC1 between weeks 12 and 24 (*F*_3, 53_ = −1.12, *p* < .01) while the subset that remained on invertebrate diet did not (*F*_3, 53_ = −0.25, *p* = .60).

**Table 1:** Innate immune function differs significantly between fruit‐ and invertebrate‐fed common bulbuls *Pycnonotus barbatus*. Differences in PC1 (decreasing haptoglobin concentration and increasing haemolysis titre), PC2 (increasing ovotransferrin concentration and haemagglutination titre) and PC3 (increasing nitric oxide concentration) between fruit‐ and invertebrate‐fed common bulbuls between week 0 and 12, and week 12 and 24. A subset of each treatment was switched to alternative diet treatment at week 12, resulting in four treatment groups: FI‐fruit to invertebrate, IF‐invertebrate to fruit, FF‐ fruit throughout and II‐invertebrate throughout

Factor	PC1			PC2		PC3	
	Haptoglobin and haemolysis			Ovotransferrin and haemagglutination		Nitric oxide	
	*df*	Chisq	*p*	Chisq	*p*	Chisq	*p*
Week 0 &12							
Sampling^a^	1	0.12	.72	**8.00**	.01**	0.27	.60
Capture^b^	1	0.24	.63	1.61	.21	0.00	.97
Sex	1	2.46	.12	0.01	.92	0.53	.47
Diet (F or I)	1	**9.48**	<.01**	**14.82**	<.01***	1.96	.16
Week	1	**16.88**	<.01***	**4.58**	.03*	0.89	.35
Diet*week	1	1.30	.25	1.79	.18	0.46	.50
Week 12 & 24							
Sampling	1	0.08	.78	**9.02**	<.01**	0.09	.76
Capture	1	0.69	.41	0.00	.99	**7.08**	<.01**
Sex	1	0.27	.61	0.00	.97	0.42	.52
Diet (FF, FI, IF or II)	3	**11.25**	<.01*	**11.79**	<.01**	5.97	.11
Week	1	0.67	.41	2.71	.10	**7.94**	<.01**
Diet*week	3	**12.85**	<.01**	3.87	.28	3.68	.30

a Sampling—time lag between capture and sampling.

b Capture—time lag between the first capture of each day and the sampling of each bird.

Statistically significant effects are highlighted bold.

*p < .05, **p < .01, ***p < .001.

**Table 2:** Post hoc summary of pairwise differences (Table 3) in PC1 (decreasing haptoglobin concentration and increasing haemolysis titre), PC2 (increasing ovotransferrin concentration and haemagglutination titre) and PC3 (nitric oxide concentration) between fruit‐ and invertebrate‐ fed subsets of common bulbuls *Pycnonotus barbatus* within week 0, 12 and 24. A subset of each treatment was switched to alternative diet treatment at week 12, resulting in four treatment groups: FI‐fruit to invertebrate, IF‐invertebrate to fruit, FF‐ fruit throughout and II‐invertebrate throughout. PC3 did not differ between treatments in weeks 0 and 12, hence no post hoc test was done

Week	Difference		PC1				PC2				PC3			
			Haptoglobin and haemolysis				Ovotransferrin and Haemagglutination				Nitric oxide			
	Diet	Diet	Est.	Error	*t*	*p*	Est.	Error	*t*	*p*	Est.	Error	*t*	*p*
Week 0 & 12														
0	I	F	−0.49	0.31	−1.59	.12	−0.59	0.31	−1.90	.06				
12	I	F	−0.96	0.32	**−3.06**	<.01**	−1.18	0.32	**−3.69**	<.01***				
Week 24														
24	II	FI	−0.07	0.50	−0.13	1.00	−0.25	0.47	−0.53	.95	0.09	0.76	0.11	1.00
24	II	FF	−0.89	0.46	−1.92	.23	−0.53	0.43	−1.23	.61	−1.16	0.71	−1.64	.35
24	II	IF	−1.10	0.44	−2.52	.07	0.10	0.41	0.25	.99	−1.74	0.68	−2.56	.05
24	FI	FF	−0.82	0.49	−1.69	.34	−0.28	0.45	−0.62	.92	−1.25	0.77	−1.62	.37
24	FI	IF	−1.04	0.46	−2.24	.13	0.35	0.43	0.82	.85	−1.83	0.76	−2.42	.07
24	FF	IF	−0.21	0.42	−0.51	.96	0.64	0.39	1.64	.35	−0.58	0.62	−0.93	.79

Statistically significant effects are highlighted bold.

**p < .01, ***p < .001.

PC2 (ovotransferrin/heamagglutination) was not different between treatment groups at the start of the experiment (Figure 2c) but was higher in fruit‐fed bulbuls compared to invertebrate‐fed ones at week 12 (Tables 1 and 2). At week 24, treatment subsets did not differ significantly from each other (Table 2), however, the invertebrate‐fed subset that was switched to fruit diet increased significantly in PC2 between weeks 12 and 24 (*F*_3, 53_ = 1.01, *p* < .01) while the other treatment subsets did not differ significantly between weeks 12 and 24 (all *p* > .3).

PC3 (nitric oxide) did not differ between diet treatment subsets at weeks 0, 12 or 24, however, the invertebrate‐fed subset switched to fruit diet increased significantly in PC3 between weeks 12 and 24 (*F*_3, 53_ = −2.08, *p* < .01) while the other treatment subsets did not differ significantly between weeks 12 and 24 (all *p* > .17).

#### 3.1.2 Body mass

**Figure 3:**
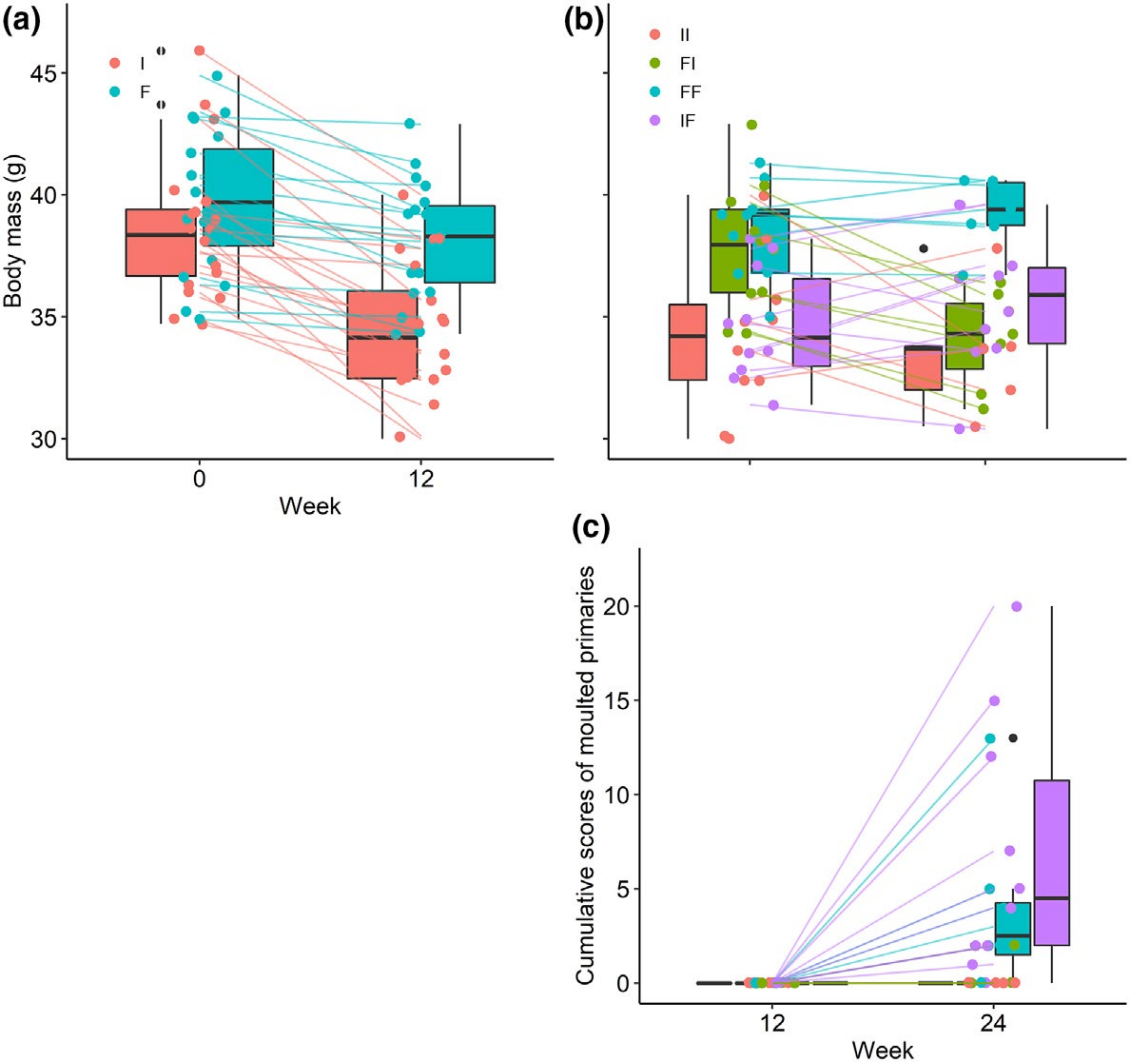
Effect of diet treatment on (a–b) body mass (g) and (c) extent of primary moult. Boxes show group median while lines connect individual birds before and after treatment. A subset of each diet treatment was switched to alternative treatment after 12 weeks. F‐fruit, I‐invertebrate, FI‐fruit to invertebrate, IF‐invertebrate to fruit, FF‐ fruit throughout and II‐invertebrate throughout. Moult commenced only after the diet switch. Black dots are outliers from box plots while coloured dots are individual points

Body mass before the experiment was similar for bulbuls later fed fruits and bulbuls later fed invertebrates (Figure 3a). However, at week 12, fruit‐fed bulbuls were significantly heavier than invertebrate‐fed ones (Tables 3 and 4). Accordingly, at week 24, the subset that was switched from invertebrate to fruits at week 12 were significantly heavier than those that remained on invertebrate diet, while those that were switched from fruit to invertebrate diet became significantly lighter than those that remained on fruits (Figure 3b, Tables 3 and 4). At week 24, treatment subsets differed significantly in body mass (Table 3). The subset switched from fruit to invertebrate diet decreased significantly in body mass between weeks 12 and 24 (*F*_3, 60_ = 2.94, *p* < .01) while the subset that remained on fruits did not (*F*_3, 60_ = −0.04, *p* = .95). The subset switched from invertebrates to fruits increased marginally in body mass between weeks 12 and 24 (*F*_3, 60_ = −1.05, *p* = .07) while the subset that remained on invertebrate diet decreased significantly (*F*_3, 60_ = 2.05, *p* < .01).

**Table 3:** Differences in body mass and extent of primary moult between fruit and invertebrate‐fed common bulbuls *Pycnonotus barbatus* between week 0 and 12 and week 12 and 24. A subset of each treatment was switched to alternative diet treatment at week 12, resulting in four treatment groups: FI‐fruit to invertebrate, IF‐invertebrate to fruit, FF‐ fruit throughout and II‐invertebrate throughout. Primary moult commenced after week 12—all birds had new or old feathers before week 12

Factor	*df*	Body mass (g)		Primary moult	
		Chisq	*p*	Chisq	*p*
Week 0 & 12					
Sampling^a^	1	0.03	.87		
Capture^b^	1	0.24	.62		
Sex	1	13.94	<.01***		
Diet (F or I)	1	7.12	.01**		
Week	1	85.08	<.01***		
Diet*Week	1	14.63	<.01***		
Week 12 & 24					
Sampling^a^		2.94	.09		
Capture^b^		0.28	.59		
Sex	1	9.20	<.01**	1.73	.19
Diet (FF, FI, IF or II)	3	18.00	<.01***	11.64	.01**
Week	1	4.69	.03*	17.75	<.01***
Diet*Week	3	23.20	<.01***	16.52	<.01***

a Sampling—time lag between capture and sample.

b Capture—time lag between the first capture of each day and the sampling of each bird.

Statistically significant effects are highlighted bold.

*p < .05, **p < .01, ***p < .001.

**Table 4:** Post hoc summary of pairwise differences (Table 1) in body mass and extent of primary moult between fruit and invertebrate‐fed subsets of common bulbuls *Pycnonotus barbatus* within week 0, 12 and 24. A subset of each treatment was switched to alternative diet treatment at week 12, resulting in four treatment groups: FI‐fruit to invertebrate, IF‐invertebrate to fruit, FF‐ fruit throughout and II‐invertebrate throughout. Primary moult commenced after week 12—all birds had new or old feathers before week 12

Week	Pairwise difference		Body mass (g)				Primary feather moult			
	Diet	Diet	Estimate	Error	*t*	*p*	Estimate	Error	*t*	*p*
Week 0 & 12										
0	I	F	−0.76	0.81	−0.94	.35				
12	I	F	−3.20	0.81	**−3.96**	<.01***				
Week 24										
24	II	FI	−1.57	1.19	−1.32	.55	−0.29	1.63	−0.18	1.00
24	II	FF	−5.72	1.22	**−4.69**	<.01***	−3.63	1.58	−2.29	.10
24	II	IF	−3.60	1.12	**−3.20**	.01**	−6.80	1.51	**−4.50**	<.01***
24	FI	FF	−4.15	1.19	**−3.47**	<.01**	−3.34	1.51	−2.21	.12
24	FI	IF	−2.03	1.06	−1.91	.22	−6.51	1.44	**−4.52**	<.01***
24	FF	IF	2.12	1.16	1.82	.26	−3.18	1.39	−2.29	.10

Statistically significant effects are highlighted bold.

**p < .01, ***p < .001.

#### 3.1.3 Extent of primary moult

Only bulbuls‐fed fruits throughout the experiment and those switched from invertebrate to fruits at week 12 had commenced primary feather moult by week 24 (Figure 3e, Tables 3 and 4). At week 24, the cumulative scores of moulted primary feathers was significantly higher compared with week 12 for the subset‐fed fruits throughout (*F*_3, 62_ = −3.63, *p* = .03) and the subset that was switched from invertebrate to fruits at week 12 (*F*_3, 62_ = −6.80, *p* < .01), but not for the subset‐fed invertebrates throughout (*F*_3, 62_ = 0, *p* = 1) and the subset that switched from fruits to invertebrates (*F*_3, 60_ = −0.29, *p* = .86).

### 3.2 Diet composition modulates covariation between body mass, moult and innate immune function

**Figure 4:**
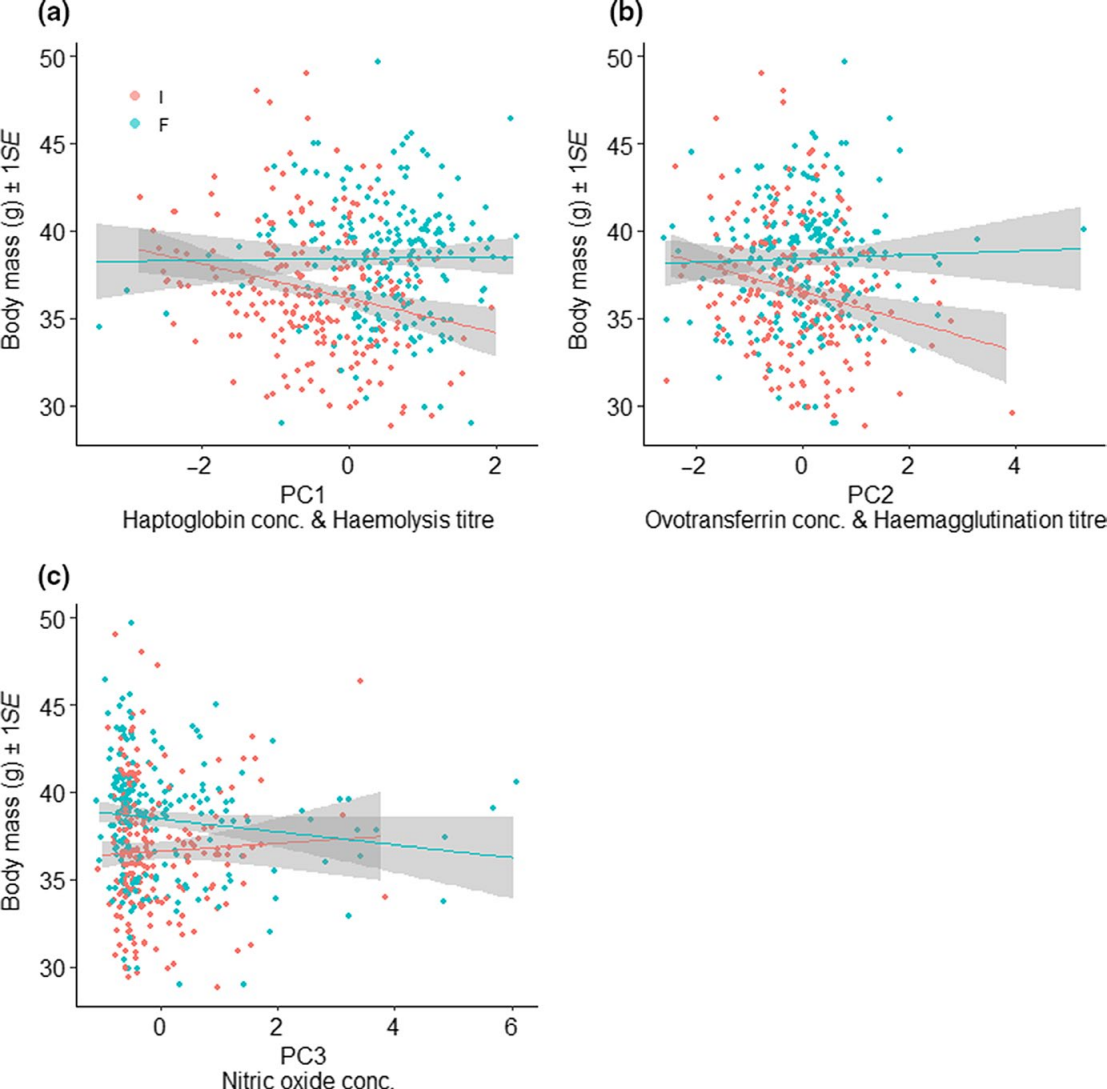
Correlation between body mass and (a) PC1 (decreasing haptoglobin concentration and increasing haemolysis titre), (b) PC2 (increasing ovotransferrin concentration and haemagglutination titre) and (c) PC3 (increasing nitric oxide concentration) in common bulbuls fed on fruit or invertebrates. Each individual was weighed and sampled before diet treatment and then subsequently, 2 weeks after restriction to invertebrate (I) or fruit (F) diet over a 24‐week period

In invertebrate‐fed bulbuls body mass decreased significantly when PC1 or PC2 increased, while for fruit‐fed bulbuls, we found no association between body mass and PC1 or PC2 (Figure 4a,b, Table 5). Body mass and PC3 were not correlated in either diet treatment groups (Figure 4c, Table 5). Body mass differed significantly among weeks, and between males and females (Table 5). PC1, PC2 and PC3 also differed significantly between weeks irrespective of diet treatment, but not between sexes (Figure S4, Table S3).

**Table 5:** Only invertebrate‐fed common bulbuls *Pycnonotus barbatus* decrease in body mass with increased immune function. Relationship between body mass and variation in PC1 (decreasing haptoglobin concentration and increasing haemolysis titre), PC2 (increasing ovotransferrin concentration and haemagglutination titre) and PC3 (increasing nitric oxide concentration) in common bulbuls fed on fruits or invertebrates. Each individual was sampled before diet treatment and then subsequently, fortnightly over a 24‐week period. Week was included to account for temporal variation in environmental factors. Individual identity and diet switching history were included as random effects to control for individual variability and treatment switch effects respectively

Factor	PC1			PC2		PC3	
	Haptoglobin and haemolysis			Ovotransferrin and haemagglutination		Nitric oxide	
	*df*	Chisq	*p*	Chisq	*p*	Chisq	*p*
Sampling^a^	1	**10.57**	<.01**	**7.62**	<.01**	**8.99**	<.01**
Capture^b^	1	**8.56**	.01**	**5.41**	.02*	**6.13**	.01*
Sex	1	**26.74**	<.01***	**25.29**	<.01***	**24.88**	<.01***
Immune function	1	**7.68**	<.01**	0.51	.47	0.04	.84
Diet	1	**20.03**	<.01***	**12.84**	<.01***	**12.95**	<.01***
Week (0–24)	11	**292.92**	<.01***	**323.57**	<.01***	**315.06**	<.01***
Immune function*Diet	1	**5.56**	.02*	**7.04**	<.01**	0.38	.54

a Sampling—time lag between capture and sampling.

b Capture—time lag between the first capture of each day and the sampling of each bird.

Statistically significant effects are highlighted bold.

*p < .05, **p < .01, ***p < .001.

**Figure 5:**
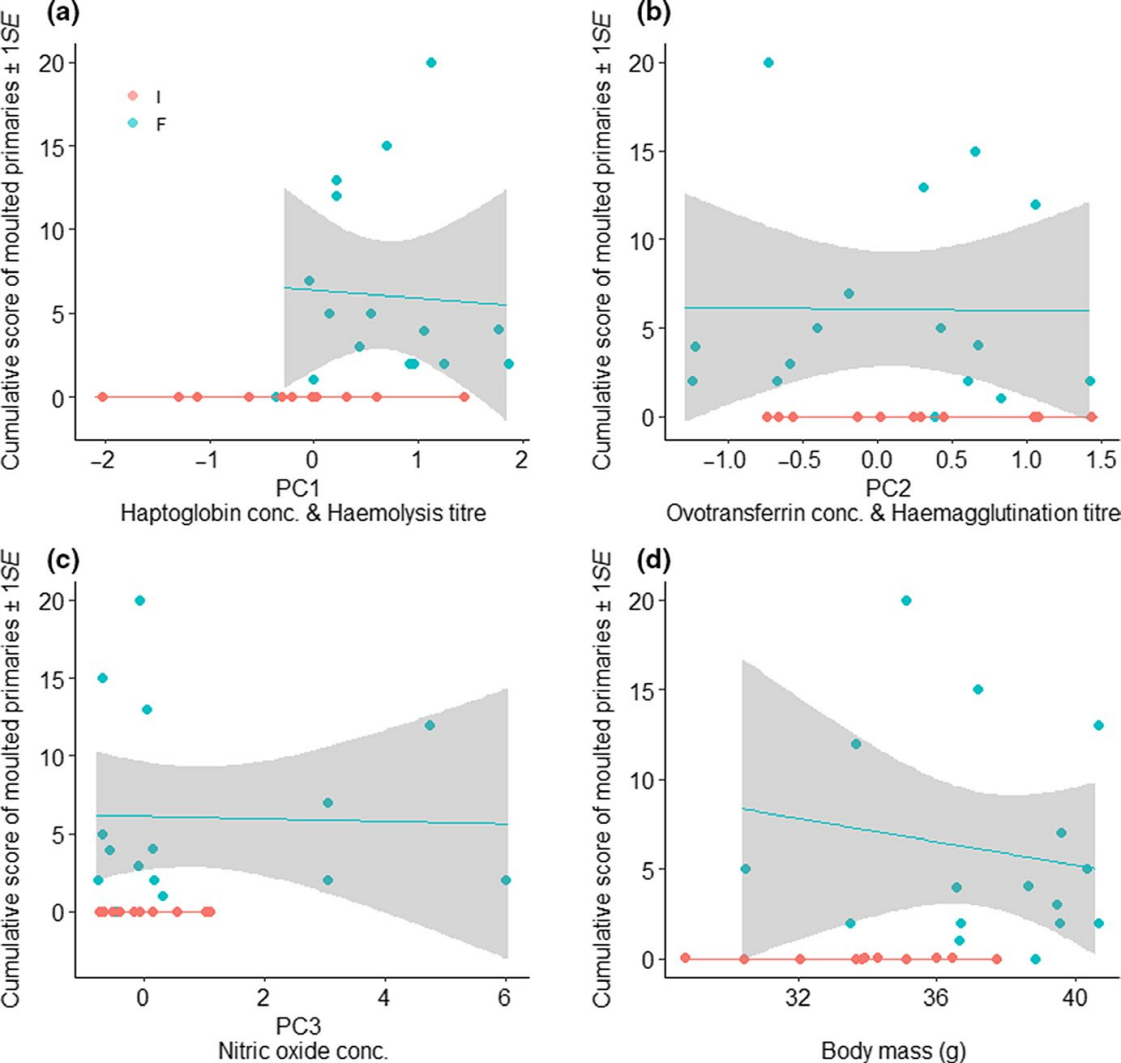
Correlation between cumulative scores of moulted primaries and (a) PC1 (decreasing haptoglobin concentration and increasing haemolysis titre), (b) PC2 (increasing ovotransferrin concentration and haemagglutination titre), (c) PC3 (increasing nitric oxide concentration) and (d) body mass (g) of common bulbuls fed on invertebrates(I) or fruits(F) for 24 weeks. Correlations for moult and immune indices obtained at week 24

Extent of primary moult was neither correlated with immune indices nor with body mass, but only fruit‐fed bulbuls had commenced moult of primary feathers at week 24 (Figure 5).

### 3.3 Diet composition more often affects body mass and immune indices directly

**Figure 6:**
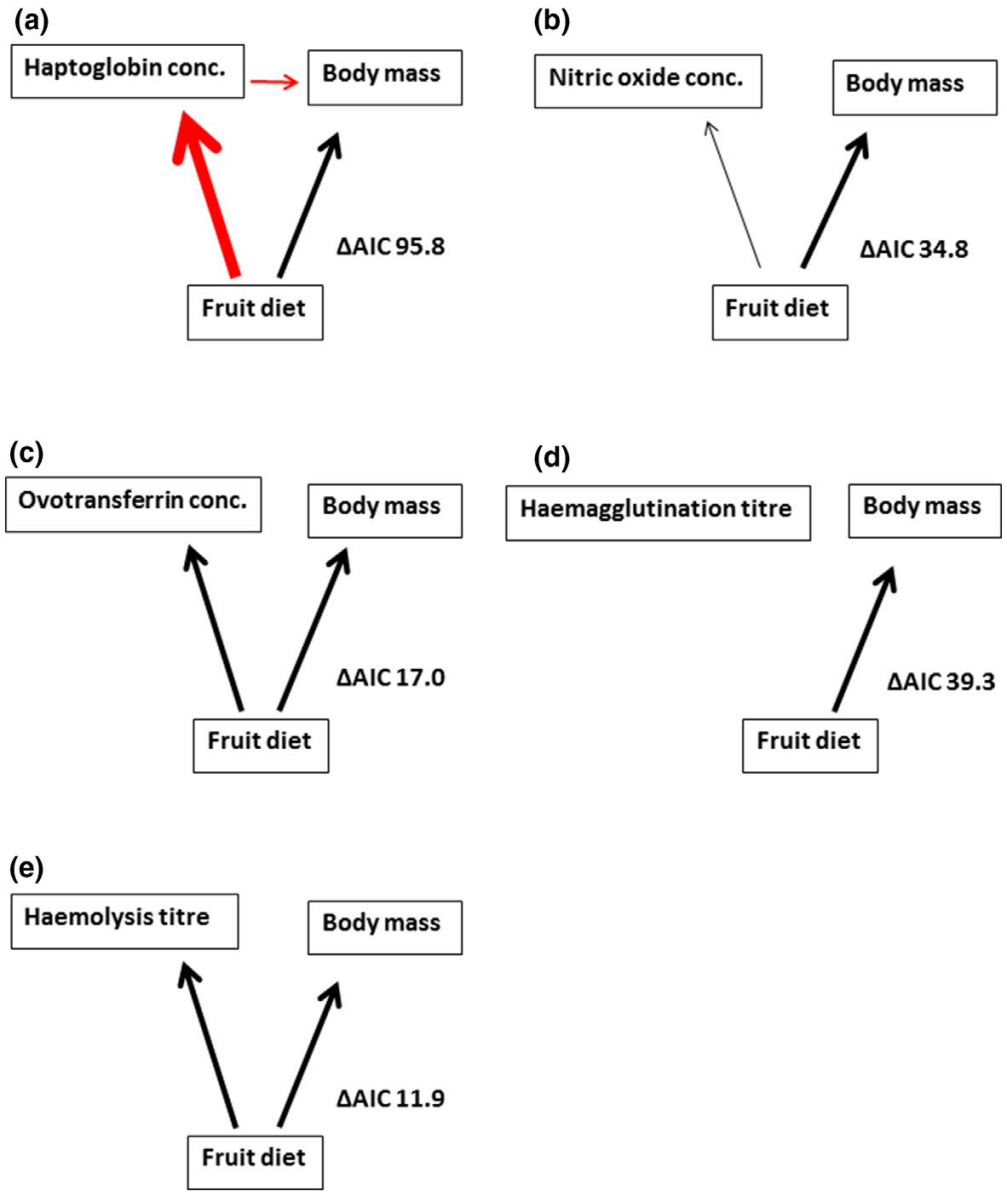
Rudimentary path diagrams showing path of diet treatment effect on body mass and immune indices ((a) haptoglobin concentration, (b) nitric oxide concentration, (c) ovotransferrin concentration, (d) haemagglutination titre and (e) haemolysis titre) in common bulbuls fed on fruit or invertebrates. Invertebrate diet was set as the model intercept. Only significant relationships are shown. Difference between the two alternative indirect pathways of diet effect tested are shown as ΔAIC (solid lines vs. dashed lines, Figure 1). Arrows indicate directionality of causal relationships. Width of arrows indicate the strength of an effect (scaled based on standardized estimates from structural equation models—Table S4). Red arrows indicate negative effects and black arrows positive effects. Overall, diet affects body mass and immune function directly for all immune indices except haemagglutination titre, but indirectly affects body mass through effect on haptoglobin concentration—body mass decreases with increased haptoglobin concentration

Diet treatment was more likely to affect immune function and body mass directly rather than indirectly via its effect on either immune function or body mass (Table S4, Figure 6a–e), although there was no evidence for a direct effect of diet treatment on haemagglutination titre (Figure 6d, *F*_1, 436_ = −0.05, *p* = .29). We only found support for an indirect effect of diet treatment on the association between haptoglobin concentration and body mass (Figure 6a). Haptoglobin concentration was significantly lower for bulbuls on a fruit diet (*F*_1, 434_ = −0.49, *p* < .01, Table S4) and correlated negatively with body mass (*F*_1, 434_ = −0.13, *p* < .01, Table S4). The association between haptoglobin concentration and body mass was more likely to be an effect of haptoglobin on body mass rather than an effect of body mass on haptoglobin concentration because the pathway—diet treatment affects haptoglobin concentration and haptoglobin concentration affects body mass was better supported than the other alternative (Figure 6a).

## 4 DISCUSSION

We found that common bulbuls fed on fruits maintained higher body mass, earlier moult and a more robust innate immune function relative to bulbuls fed on invertebrates. This was also reflected in the absence of body mass loss with higher immune response and lower haptoglobin concentrations in the fruit‐fed birds. Invertebrate‐fed birds on the other hand decreased body mass with higher immune response and had higher haptoglobin concentration. Exploration of the mechanistic connection between immune indices and body mass revealed that diet treatment was more likely to affect body mass and immune indices directly rather than via alternative indirect pathways. The only exceptions were haemagglutination titre which was not significantly affected by diet treatment, and haptoglobin concentration which was associated with loss of body mass.

We found no indication that the fruit‐fed bulbuls—that presumably suffered protein limitation—had impaired innate immune function, body mass or moult as we predicted (Lochmiller, Vestey, & Boren, 1993). On the contrary, bulbuls were in better condition when fed on fruits than invertebrates, and this was reversible. Apparently, essential amino acids from the invertebrate diet are not the limiting nutrients for maintenance of immune function, body mass and moult in Common Bulbuls. The occurrence of lower body mass and absence of moult, coupled with lower indices of immune function combined, but higher haptoglobin concentrations specifically, in invertebrate‐fed bulbuls indicates poorer health (Owen‐Ashley & Wingfield, 2006). Note however, that bulbuls with the lowest body masses, maintained weight well above the lower threshold of the body mass range of wild bulbuls, 25–50 g.

The mechanism generating the opposite response in fruit‐fed bulbuls is not clear. A striking difference between the two diets is the presence of carotenoids and sugars in fruits and their absence in invertebrates. Carotenoids and sugars have been proposed to function as antioxidants (Griffiths et al., 2016; Levin, Lopez‐Martinez, Fane, & Davidowitz, 2017), and therefore, may have immune‐modulatory and anti‐inflammatory effects (Chew & Park, 2004). Carotenoids especially, modulate a range of immune parameters, including stimulating T‐cell and antibody production, and reducing oxidative damage during immune response (Chew & Park, 2004). While the lower haptoglobin concentration may be linked to the anti‐inflammatory role of carotenoids (Park, Chyun, Kim, Line, & Chew, 2010) in fruit‐fed bulbuls, the exact links between carotenoids and the other immune indices we measured are not known. Carotenoids are often considered vital for immune function because their deficiency in diet is associated with dulling of carotenoid‐based pigmentation during infection (Baeta, Faivre, Motreuil, Gaillard, & Moreau, 2008; Torres & Velando, 2007). However, compounds such as flavonoids, vitamins, iodine, fluorine and specific fatty acids (Demas, Greives, Chester, & French, 2012) and the gut microbiome (Belkaid & Hand, 2014) are also vital for immune function and may differ in composition between fruit and invertebrate‐fed bulbuls.

We do not know whether the invertebrate‐fed bulbuls became infected by new agents or became more susceptible to already harboured infections, but because their natural antibody and complement activities were lower (PC1 & PC2), they may be more prone to infection (Ochsenbein et al., 1999). Natural antibodies function as recognition molecules capable of opsonizing invading microbes and initiating a complement enzyme cascade, which ends in the destruction of the invading microbe (Belperron & Bockenstedt, 2001; Reid, Prodeus, Khan, Hsu, & Rosen, 1997). Their activity forms a first line of defense and a useful link between innate and adaptive immunity (Ochsenbein & Zinkernagel, 2000; Panda & Ding, 2015; Schmid‐Hempel & Ebert, 2003). We propose that the observed direct effect of diet composition on immune indices, body mass and moult may reflect a cascade of events: fruit deprivation possibly impaired prophylactic components of innate immunity (such as natural antibody and compliment activities which clear early stages of infection), leading to increased inflammation, loss of body mass and ultimately delayed moult. Nonetheless, omnivory seems facultative rather than obligate because bulbuls survived temporarily on either diet. But prolonged fruit deprivation was clearly more detrimental than invertebrate deprivation, because seven of nine birds that died during the experiment (Table S1) were on an invertebrate diet and deteriorated significantly in condition during the second half of the experiment. The two birds that died from the fruit treatment were due to accidents.

Our results confirm that immunological resource requirements are precise‐specific nutrients rather than energy (Cotter et al., 2011; Demas et al., 2012; Klasing, 2007; Ponton et al., 2013): this is clearly reflected in the difference between diet treatments with ad libitum food supply. Immune function may require large amounts of specific nutrients, for example, acute phase response may result in about six‐fold increase in lysine utilization (Klasing, 2007) while immune activation by lipopolysaccharide and phytohaemagglutanin depletes retinal carotenoid (Toomey, Butler, & McGraw, 2010). It is unlikely that temporal diet shifts are merely due to variation in food availability, in birds, they are primarily a result of active changes in food preferences corresponding to changing nutritional requirements which have survival consequences (Bairlein, 1996). Therefore, it seems likely that it is nutrient deficiency for the immune system that impairs immune function (Siva‐Jothy & Thompson, 2002) rather than frugivory or insectivory. Animals have been shown to select food items which facilitate recovery during infection (Povey, Cotter, Simpson, Lee, & Wilson, 2009; Povey, Cotter, Simpson, & Wilson, 2014) and this may be related to the specific nutrient requirements for immune response. Differences in energy content of food may affect immune function, but the difference in energy content between the fruit and invertebrate diet is unlikely to be the cause of the observed difference in this experiment, because protein‐based diets, such as the invertebrate diet, are higher in energy per unit mass compared to fruits (Barker, Fitzpatrick, & Dierenfeld, 1998; Friedman, 2008). Moreover, because food was provided ad libitum, birds could increase food, and thus energy intake if needed, albeit with adjustments to their digestive systems (McWilliams & Karasov, 2001; Piersma et al., 1999).

The digestive systems of animals need to be adapted to a diet to optimize digestion, and thus nutrient and energy uptake (Karasov, Martínez del Rio, & Caviedes‐Vidal, 2011). There is no indication that a lower digestibility of invertebrates led to the observed differences in immune function, body mass and moult in common bulbuls because 12 weeks, the first half of the experiment, was long enough for adjustment of the digestive system. Short‐term adjustments range from enlargement of the gut to allow processing of larger amounts of lower quality food to production of specific enzymes or alteration of the gut microbiome to facilitate digestion and assimilation (Ciminari, Afik, Karasov, & Caviedes‐Vidal, 2001; Karasov et al., 2011). In American Robins *Turdus migratorius* and European Starlings *Sturnus vulgaris* switching between fruit and insects, these adjustments take about 7–10 days (Levey & Karasov, 1989). If common bulbuls were unable to enlarge their digestive tract sufficiently to process large amounts of lower energy food at once, they could forage on smaller amounts of such food items over a longer period time (Karasov, Phan, Diamond, & Carpenter, 1986) since foraging time was not restricted and so, can still maximize energy uptake. It is unlikely that common bulbuls lacked both the capacity to process larger amounts of invertebrates at once or intermittently to increase energy uptake. Animals may however, gain sufficient amount of energy and yet limited amount of required nutrients if these are unavailable or inaccessible, and this may be the case for the common bulbuls. Although fruits are generally less digestible than invertebrates due to lower gut retention time and high fibre content (Afik & Karasov, 1995; Levey & Duke, 1992) fruits provided better nutrition.

Long‐term adaptation to a diet due to phylogeny (Karasov & Douglas, 2013) may likely be important for bulbuls. The majority of the family Pycnonotidae to which the Common Bulbul belongs are frugivores that supplement their diet with insects (Fishpool & Tobias, 2005). Being more frugivorous than insectivorous may result in a better capacity to harness nutrients from fruits than invertebrates and this may partly account for the better performance of birds on a fruit diet, assuming that fruits are the optimal diet for Common Bulbuls. It still remains interesting however, that immune function, body mass and moult were all better supported by a protein poor diet, because in other taxa, for example, insects, individuals recovering from infection prefer protein‐rich diets (Povey et al., 2014).

The co‐occurrence of decreased body mass with higher immune response and delayed moult was only present in invertebrate‐fed bulbuls, not in fruit‐fed bulbuls, and may indicate a diet‐dependent trade‐off between these traits. The contrasting pattern between treatments suggests that disease susceptibility increases when resources are insufficient to sustain optimal immunity (Cornet, Bichet, Larcombe, Faivre, & Sorci, 2014; Nelson, Demas, Klein, & Kriegsfeld, 2002). Previous experiments that used similar immune indices to our study, but restricting food availability rather than nutrients, found no evidence for downregulation of constitutive innate immune function (Buehler et al., 2009; Schultz, Hahn, & Klasing, 2017) in food restricted birds. We therefore hypothesize, that under low energy budgets, common bulbuls may still maintain aspects of innate immune function (Hegemann, Matson, Versteegh, & Tieleman, 2012) if required nutrients are sufficiently available. However, under specific nutrient limitation, they may prioritize pathogen defence by trading‐off body mass and moult to free resources for acute phase response, because disease presents an immediate risk to death (WHO, 2018). Innate immunity is thus, a component of body condition that is associated with the maintenance of body mass and onset of annual moult—a key life‐history event for common bulbuls (Nwaogu, 2019; Nwaogu, Tieleman, et al., 2019). However, the extent of moult in fruit‐fed bulbuls was not correlated with body mass or immune function, even though invertebrate‐fed bulbuls in poorer condition did not moult until the end of the experiment, suggesting that it is moult initiation and maintenance that is condition‐dependent not moult extent (Murphy & King, 1992).

We found that immune indices and body mass were most likely affected directly by diet treatment. The only exceptions were haemagglutination titre which was not affected by diet treatment, and haptoglobin concentration which was negatively correlated to body mass. Note however, that diet composition affected haemagglutination titre during the first 12 weeks of the experiment (combined with ovotransferrin as PC1), thus the absence of a treatment effect in the full range of the experiment may be associated with increased variability in immune indices (Figure S3) observed after the onset of the rains (Figure S1). In the wild, innate immune function differs between the wet and dry season in common bulbuls (Nwaogu, Cresswell, Versteegh, & Tieleman, 2019; Nwaogu, 2019). The negative correlation between haptoglobin concentration and body mass may be due to a breakdown of muscle proteins to supply amino acids for hepatic acute phase protein synthesis (Jain et al., 2011; Londhe & Guttridge, 2015). Curiously, ovotransferrin concentration was not associated with body mass variation and showed an opposite trend with haptoglobin concentration despite also being an acute phase protein. The reason for this might be that unlike haptoglobin, ovotransferrin is a negative acute phase protein which decreases with high inflammation because temporarily high free hormones may be bound to ovotransferrin during inflammation. Furthermore, haptoglobin may be produced at the expense of ovotransferrin (Giansanti et al., 2012; Gruys et al., 2005). The opposite trend between haptoglobin concentration and other immune indices suggest connections among immune indices, and this may result from cascading processes or trade‐offs within the immune system. Innate immune indices may have adaptive features (Kvell, Cooper, Engelmann, Bovari, & Nemeth, 2007)—one layer of immune defence may not be required until another is initiated, suppressed or surpassed (Ochsenbein et al., 1999; Panda & Ding, 2015). Our results show unequivocally that high lytic capacity covaries with low haptoglobin concentration in healthier common bulbuls (PC1), demonstrating that depending on the immune index considered, immune function may vary positively, negatively, or show no association with body mass as proxy of general physical condition or other life‐history traits such as moult. Experimental studies testing trade‐offs between immune function and other life‐history traits should aim at manipulating inflammatory response or nutrient availability because manipulations targeted at physical condition in breeding animals, for example, may be transferred to offspring (Tieleman, Dijkstra, Klasing, Visser, & Williams, 2008) or only visible in the long term (Hegemann, Matson, Flinks, et al., 2013a). Disparities between studies that manipulated inflammatory response may have arisen from differences in resource constraints (Råberg, Nilsson, Ilmonen, Stjernman, & Hasselquist, 2000; Williams, Christians, Aiken, & Evanson, 1999), behavioural adjustments (Ardia, 2005b) or individual quality (Ardia, 2005a). Nonetheless, the extent of variation within the immune system is far from being fully understood.

Besides confirming that innate immunity is nutrient specific (Klasing, 1998, 2007), this study is unique because we show a reversible effect of diet composition on wild adult birds whose immune systems are presumably fully developed and adapted to wild conditions—demonstrating a short‐term consequence of diet alteration on the life‐history traits of animals (Siva‐Jothy & Thompson, 2002). A crucial next step is decomposing dietary components to identify specific functions, and possibly the role of the gut microbiome. We propose that seasonal diet composition plays an important role in maintaining seasonal variation in immune function (Durand & Morel, 2008; Hegemann, Matson, Versteegh, et al., 2013b), allowing animals to combat seasonal immune challenges. Therefore, if life‐history events coincide with sufficient availability of required nutrients, trade‐offs between immune function and life‐history events (Hasselquist & Nilsson, 2012; Sheldon & Verhulst, 1996) can be avoided. This principle may explain the strict seasonal timing of life‐history events in seasonal environments and the blurring of seasonal patterns in life‐history events in environments with mild resource seasonality (Merrill et al., 2015).

## AUTHORS' CONTRIBUTIONS

C.J.N., B.I.T. and W.C. designed and raised funds. C.J.N. and A.G. were responsible for aviary experiments, while B.I.T., M.W.D. and W.C. supervised. C.J.N., A.G., B.I.T., M.W.D. and W.C. analysed data and interpreted results. C.J.N. developed the first draft of the manuscript. All authors read and approved manuscript.

## Data Availability

Data are deposited in Dryad Digital Repository: https://doi.org/10.5061/dryad.bg79cnp77 (Nwaogu, Galema, Cresswell, Dietz, & Tieleman, 2019).
