# Supplementary material for: A fruit diet rather than invertebrate diet maintains a robust innate immunity in an omnivorous tropical songbird
Source: J Anim Ecol. 2020 Jan 3;89(3):867–83. doi: 10.1111/1365-2656.13152 (PMC7079115; doi:10.1111/1365-2656.13152)
Supplement: Supplementary file 1 [file JANE-89-867-s001.docx]

**SUPPLEMENTARY INFORMATION**

**A fruit diet rather than invertebrate diet maintains a robust innate immunity in an omnivorous tropical songbird**

Chima J. Nwaogu^1, 2, 3^*^†^, Annabet Galema^1^, Will Cresswell^2, 3^, Maurine W. Dietz^1^ and B. Irene Tieleman^1^

^1^Groningen Institute for Evolutionary Life Sciences, University of Groningen, P.O. Box 11103, 9700 CC, Groningen, The Netherlands.

^2^School of Biology, University of St Andrews, Harold Mitchell Building, St Andrews Fife KY16 9TH, UK.

^3^ A.P. Leventis Ornithological Research Institute, Jos, Nigeria.

*Corresponding author: [c.j.nwaogu@rug.nl](mailto:c.j.nwaogu@rug.nl)

^†^Corresponding author’s current address: Fitzpatrick Institute of African Ornithology, University of Cape Town, 7701, Rondebosch, Cape Town, South Africa.

**Study species and environmental conditions in study site**

We study Common Bulbuls *Pycnonotus barbatus* in the Amurum Forest Reserve (09°52’N, 08°58’E) at the A.P. Leventis Ornithological Research Institute on the Jos Plateau in north central Nigeria. The reserve is a heterogeneous woodland habitat with inselbergs and interspersed riparian forests, surrounded by farmlands and human settlements. It experiences a single wet and dry season annually. The wet season usually lasts from April to October. Minimum and maximum daily temperatures vary in a bimodal fashion due to increased cloud cover in the wet season and the occurrence of cold dry north-easterly trade winds during the periods between November and February.

Common bulbuls are 25 – 50g sexually monomorphic resident passerines. In central Nigeria, they breed year-round but with small breeding peaks at the end of the dry season/start of the wet season and the end of the wet season (Nwaogu et al. 2019). Moult is seasonal and takes place in the wet season for most individuals. Males moult about 21 days earlier than females, but on average moult starts on the 1^st^ of May annually at the population level (Nwaogu et al. 2019).

Common Bulbuls feed largely on fruits and insects, although nestlings are predominantly fed insects and then fruits later. Adult birds also feed occasionally on nectar or seeds. Generally, fruits and insects are available year-round, because some plants flower and fruit during the dry season, attracting large numbers of insects and birds. A few gullies in riparian forest fragments retain water even in the dry season and provide drinking water for animals and nourishment for plants. Therefore, a combination of environmental heterogeneity, plant phenology and distribution, ensure year-round omnivory in the Common Bulbul, although with some seasonal variability in abundance of different food items.

**Reference**

Nwaogu, C. J., Tieleman, B. I. and Cresswell, W. 2019. Weak breeding seasonality of a songbird in a seasonally arid tropical environment arises from individual flexibility and strongly seasonal moult. - Ibis 161: 533–545.

**Experimental set up**

Invertebrate

Fruits

10 birds

10 birds

10 birds

10 birds


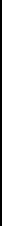

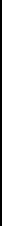


Week 1

Week 12

Week 24

**Figure S1**: Experimental set-up – 10 of 20 Common Bulbuls in each diet treatment were switch to alternative treatments 12 weeks after diet manipulation, while the other 10 of each treatment were maintained on the same treatment until week 24. Birds were housed in four aviaries, two aviaries were supplied fruits while the other two were supplied invertebrates, hence, five birds from each aviary were switched to the alternative diet treatment while the other 5 were maintained on the same diet. Five of each group of 10 birds indicated in the figure (above) were housed in different aviaries. Switched birds from the fruit treatment replaced those switched from invertebrate to fruit treatment and vice versa, so we maintained four aviaries with the same diet treatment throughout the experiment, allowing all birds to change social groups half-way into the experiment.

**Table S1:** Sample sizes and sex-mix of captive wild caught Common Bulbuls *Pycnonotus barbatus* maintained on experimental diet manipulation between weeks 0 and 24. The overall reduction in sample size was due to nine recorded mortalities and one bird escaping from captivity during the experiment. Seven birds from the invertebrate treatment died during the second half of the experiment after a deterioration of physical condition, while two birds from the fruit treatment died from sampling accidents.

| **Week** |  | **0** | **2** | **4** | **6** | **8** | **10** | **12** | **14** | **16** | **20** | **22** | **24** |
| --- | --- | --- | --- | --- | --- | --- | --- | --- | --- | --- | --- | --- | --- |
| **Fruit** | **Female** | 6 | 6 | 6 | 6 | 6 | 6 | 6 | 8 | 7 | 6 | 6 | 6 |
|  | **Male** | 14 | 13 | 13 | 13 | 13 | 13 | 13 | 11 | 11 | 11 | 11 | 11 |
|  | **Total** | **20** | **19** | **19** | **19** | **19** | **19** | **19** | **19** | **18** | **17** | **17** | **17** |
|  |  |  |  |  |  |  |  |  |  |  |  |  |  |
| **Invertebrate** | **Female** | 9 | 9 | 9 | 9 | 9 | 9 | 9 | 7 | 7 | 7 | 7 | 5 |
|  | **Male** | 11 | 11 | 11 | 11 | 11 | 11 | 11 | 12 | 12 | 11 | 10 | 8 |
|  | **Total** | **20** | **20** | **20** | **20** | **20** | **20** | **20** | **19** | **19** | **18** | **17** | **13** |

**Figure S2**: Natural variation in environmental condition and body mass over the time course of the diet experiment: Daily rainfall (mm) (A), temperature range (°C) (B), minimum temperature (°C) (C), maximum temperature (°C) (D) and body mass (g) of captive Common Bulbuls during diet experiment (E). body mass (g) of wild Common Bulbuls (F) from December 2014 to May 2015, corresponding to the period of diet experiment in 2016/2017. Weather data was obtained from the Nigerian Metrological Agency at the Jos Airport located 26km from the A. P. Leventis Ornithological research Institute.

**Table S2**: Principal component axes loadings of five innate immune indices. PC1 represents decreasing haptoglobin concentration and increasing haemolysis titre, PC2 represents increasing ovotransferrin concentration and haemagglutination titre, while PC3 represents increasing nitric oxide concentration.

| **Original variables** | **PC1** | **PC2** | **PC3** |
| --- | --- | --- | --- |
| Ovotransferrin concentration | 0.188 | **0.736** |  |
| Nitric oxide concentration |  |  | **0.992** |
| Haptoglobin concentration | **-0.742** | 0.168 |  |
| Haemagglutination titre | -0.151 | **0.722** |  |
| Haemolysis titre | **0.742** | 0.206 |  |

**Figure S3**: Variation in innate immune function over 24 weeks of diet restriction to fruits or invertebrates in Common Bulbuls *Pycnonotus barbatus*: PC1 (decreasing haptoglobin and increasing haemolysis titre), PC2 (increasing ovotransferrin concentration and haemagglutination titre) and PC3 (increasing nitric oxide concentration). A subset of each treatment group was switched to alternative diet treatment after sampling at week 12. F-fruit, I-invertebrate, FI-fruit to invertebrate, IF-invertebrate to fruit, FF- fruit throughout and II-invertebrate throughout.

**Figure S4**: Temporal variation in body mass (g) over 24 weeks of diet restriction to fruits or invertebrates in Common Bulbuls *Pycnonotus barbatus*. A subset of each diet treatment group was switched to alternative diet after sampling at week 12. F-fruit, I-invertebrate, FI-fruit to invertebrate, IF-invertebrate to fruit, FF- fruit throughout and II-invertebrate throughout.

**Table S3**: Innate immune function differs significantly between weeks in Common Bulbuls *Pycnonotus barbatus* irrespective of diet treatment. Individual identity and diet switch history were included as random factors to control for individual variability and treatment effects, respectively.

| **PC1 - Haptoglobin and Haemolysis** | | | | |
| --- | --- | --- | --- | --- |
| **Factor** | **Df** | **Chisq** | **P** |  |
| Sampling | 1 | 2.43 | 0.12 |  |
| **Capture** | **1** | **5.62** | **0.02** | ***** |
| **Diet** | **1** | **105.07** | **< 0.01** | ******* |
| **Week** | **11** | **50.39** | **0.00** | ******* |
| **Body mass** | **1** | **7.56** | **0.01** | ****** |
| **Sex** | **1** | **6.70** | **0.01** | ****** |
| Diet:Week | 11 | 12.64 | 0.32 |  |
|  |  |  |  |  |
| **PC2 - Ovotransferin and Haemagglutination** | | | | |
| **Factor** | **Df** | **Chisq** | **P** |  |
| Sampling | 1 | **3.91** | **0.05** | * |
| Capture | 1 | 0.37 | 0.54 |  |
| Diet | 1 | 2.49 | 0.11 |  |
| Week | 11 | **22.45** | **0.02** | * |
| Body mass | 1 | 0.65 | 0.2 |  |
| Sex | 1 | 0.23 | 0.63 |  |
| Diet:Week | 11 | 13.02 | 0.29 |  |
|  |  |  |  |  |
| PC3 - Nitric oxide | | | | |
| **Factor** | **Df** | **Chisq** | **P** |  |
| Sampling | 1 | 0.34 | 0.56 |  |
| Capture | 1 | 2.18 | 0.14 |  |
| **Diet** | **1** | **5.71** | **0.02** | ***** |
| **Week** | **11** | **23.78** | **0.01** | ***** |
| Body mass | 1 | 0.01 | 0.90 |  |
| Sex | 1 | 0.03 | 0.87 |  |
| Diet:Week | 11 | 17.09 | 0.11 |  |

**^1^Sampling** - time lag between capture and sampling

^2^**Capture** – time lag between the first capture of each day and the sampling of each bird

**Table S4:** Summarised output from structural equation models testing alternative path of diet treatment effect on body mass and immune function in Common Bulbuls fed on fruits or invertebrates. Models and AICs highlighted bold indicate selected structural models explaining the most likely paths of diet treatment effect on body mass and/or specific immune indices (see Fig. 1). Haptoglobin concentration is the only immune index that is significantly associated with body mass variation, otherwise diet treatment affects immune indices and body mass directly, except for haemagglutination titre which was not affected by diet treatment.

|  | HP |  |  |  |  |  |  |  |
| --- | --- | --- | --- | --- | --- | --- | --- | --- |
| S-Model | AIC | L-Model | Response | Predictor | DF | Std Est. | P |  |
|  |  | 1 | Hp | DietF | 434 |  | <0.01 | *** |
| A | 150.0 |  | Mass | DietF | 435 | 0.26 | <0.01 | *** |
|  |  | 2 | Hp | Mass | 435 | -0.13 | 0.01 | * |
|  |  |  |  |  |  |  |  |  |
|  |  | 1 | Mass | Diet | 434 |  | <0.01 | *** |
| B | 54.2 |  | Hp | DietF | 436 | -0.49 | <0.01 | *** |
|  |  | 2 | Mass | Hp | 435 | -0.13 | <0.01 | ** |
|  | NOX |  |  |  |  |  |  |  |
|  |  | 1 | Nox2 | DietF | 425 |  | 0.04 | * |
| A | 26.6 |  | Mass | DietF | 426 | 0.25 | <0.01 | *** |
|  |  | 2 | Nox2 | Mass | 426 | -0.03 | 0.52 |  |
|  |  |  |  |  |  |  |  |  |
|  |  | 1 | Mass | DietT | 425 |  | <0.01 | *** |
| B | 61.4 |  | Nox2 | DietF | 426 | 0.09 | 0.07 |  |
|  |  | 2 | Mass | Nox2 | 426 | -0.01 | 0.74 |  |
|  | OVO |  |  |  |  |  |  |  |
|  |  | 1 | Ovo | DietT | 406 |  | <0.01 | *** |
| A | 42.5 |  | Mass | DietF | 407 | 0.26 | <0.01 | *** |
|  |  | 2 | Ovo | Mass | 407 | 0.01 | 0.90 |  |
|  |  |  |  |  |  |  |  |  |
|  |  | 1 | Mass | DietT | 407 |  | <0.01 | *** |
| B | 59.5 |  | Ovo | DietF | 407 | 0.20 | <0.01 | *** |
|  |  | 2 | Mass | Ovo | 5 | 0.00 | 0.98 |  |
|  | AGG |  |  |  |  |  |  |  |
|  |  | 1 | Agg | DietT | 434 |  | 0.16 |  |
| A | 23.7 |  | Mass | DietTF | 435 | 0.26 | 0.00 | *** |
|  |  | 2 | Agg | Mass | 435 | -0.06 | 0.25 |  |
|  |  |  |  |  |  |  |  |  |
|  |  | 1 | Mass | DietF | 434 |  | <0.01 | *** |
| B | 63.0 |  | Agg | DietF | 436 | 0.05 | 0.29 |  |
|  |  | 2 | Mass | Agg | 435 | -0.03 | 0.52 |  |
|  | Lysis |  |  |  |  |  |  |  |
|  |  | 1 | Lys | DietF | 434 |  | <0.01 | *** |
| A | 50.4 |  | Mass | DietF | 435 | 0.26 | 0.00 | *** |
|  |  | 2 | Lys | Mass | 435 | 0.04 | 0.47 |  |
|  |  |  |  |  |  |  |  |  |
|  |  | 1 | Mass | DietF | 434 |  | <0.01 | *** |
| B | 62.3 |  | Lys | DietF | 436 | 0.23 | <0.01 | *** |
|  |  | 2 | Mass | Lys | 435 | 0.04 | 0.41 |  |
